# Transcriptomic analysis of staphylococcal sRNAs: insights into species-specific adaption and the evolution of pathogenesis

**DOI:** 10.1099/mgen.0.000065

**Published:** 2016-07-26

**Authors:** William H. Broach, Andy Weiss, Lindsey N. Shaw

**Affiliations:** Department of Cell Biology, Microbiology and Molecular Biology, University of South Florida, Tampa, FL 33620, USA

**Keywords:** sRNA, small regulatory RNA, *Staphylococcus aureus*, *Staphylococcus epidermidis*, *Staphylococcus carnosus*, transcriptomics, RNA sequencing, RNAseq, genome annotation, comparative genomics, RNA structure

## Abstract

Next-generation sequencing technologies have dramatically increased the rate at which new genomes are sequenced. Accordingly, automated annotation programs have become adept at identifying and annotating protein coding regions, as well as common and conserved RNAs. Additionally, RNAseq techniques have advanced our ability to identify and annotate regulatory RNAs (sRNAs), which remain significantly understudied. Recently, our group catalogued and annotated all previously known and newly identified sRNAs in several *Staphylococcus aureus* strains. These complete annotation files now serve as tools to compare the sRNA content of *S. aureus* with other bacterial strains to investigate the conservation of their sRNomes. Accordingly, in this study we performed RNAseq on two staphylococcal species, *Staphylococcus epidermidis* and *Staphylococcus carnosus*, identifying 118 and 89 sRNAs in these organisms, respectively. The sRNA contents of all three species were then compared to elucidate their common and species-specific sRNA content, identifying a core set of between 53 and 36 sRNAs encoded in each organism. In addition, we determined that *S. aureus* has the largest set of unique sRNAs (137) while *S. epidermidis*has the fewest (25). Finally, we identify a highly conserved sequence and structural motif differentially represented within, yet common to, both *S. aureus* and *S. epidermidis.* Collectively, in this study, we uncover the sRNome common to three staphylococcal species, shedding light on sRNAs that are likely to be involved in basic physiological processes common to the genus. More significantly, we have identified species-specific sRNAs that are likely to influence the individual lifestyle and behaviour of these diverse staphylococcal strains.

## Data Summary

1. The RNAseq results have been deposited to the NCBI Gene Expression Omnibus; GEO submission GSE77567 (url – http://www.ncbi.nlm.nih.gov/geo/query/acc.cgi?token=olgjaisktxerfqz&acc=GSE77567).
2. The updated GenBank file, containing novel sRNA annotations (annotated as ‘misc. RNA') has been deposited to Figshare; DOI: 10.6084/m9.figshare.3385861 (url – https://figshare.com/s/ac122d912782908e6359).

## Impact Statement

*Staphylococcus aureus* is a leading cause of nosocomial infections and exhibits profound levels of antimicrobial resistance. The importance of this pathogen has been well established, forming the subject of extensive research, but a comprehensive understanding of the regulatory processes governing its virulence has yet to be elucidated. Recently, our group has investigated the role of regulatory RNAs (sRNAs) by cataloguing and annotating them in *S. aureus* genomes. The study presented here continues this line of research by performing transcriptomic analyses with two closely related species, *Staphylococcus epidermidis* and *Staphylococcus carnosus*, to annotate, for the first time, their genomes for sRNAs. The sRNAs of all three organisms were then compared to determine the common and species-specific sRNA content of each genome. In addition, we identified a subset of sRNAs shared between *S. aureus* and *S. epidermidis* that demonstrate high sequence and structural conservation. This study provides a platform to guide studies on sRNAs that are important for the general physiology of staphylococci (shared sRNAs) as well as the unique lifestyles of each organism (species-specific sRNAs).

## Introduction

The wide availability of sequenced genomes and the decreasing cost of producing such data have revolutionized the way molecular biology research is performed (Dark, 2013). With the increasing knowledge base of genomic information published each year there is an escalating demand for automated pipelines to identify and annotate genes within sequence data (Richardson & Watson, 2013). To highlight the vast amount of genetic information available, at the time of writing this manuscript, a total of 5443 completed prokaryotic genomes were available in the NCBI Genome database (http://www.ncbi.nlm.nih.gov/genome), with a further 65,259 partially completed genomes. Furthermore, the rate of publication continues to increase exponentially each year for studies on such topics (Tatusova *et al.*, 2015). Traditionally, the pipelines used for *de novo* genome assembly involve prediction of protein-coding genes, rRNAs and tRNAs, followed by comparison with a reference genome to assign ORF function (Richardson & Watson, 2013). However, many drawbacks exist to such approaches, not the least of which is a lack of efficient detection for small, regulatory RNAs (sRNAs), some of which can also encode small peptides (<50 aa).

sRNAs as a class of molecule are increasingly recognized as playing important regulatory roles in bacteria (Beisel & Storz, 2010; Murphy *et al*., 2014; Caron *et al.*, 2010; Geissmann *et al.*, 2006; Harris *et al.*, 2013; Hoe *et al.*, 2013; Weiberg *et al.*, 2015; Papenfort & Vanderpool, 2015; Oliva *et al.*, 2015; Papenfort & Vogel, 2014; Weilbacher *et al.*, 2003). For example, sRNAs regulate a wide variety of cellular processes, such as carbon metabolism and iron acquisition, and also have profound influence on virulence gene expression in many important pathogenic bacteria (Geissmann *et al.*, 2006; Oliva *et al.*, 2015; Caswell *et al.*, 2012; Giangrossi *et al.*, 2010; Broach *et al*., 2012; Hoe *et al.*, 2013; Beisel & Storz, 2010; Weilbacher *et al.*, 2003; Papenfort & Vogel, 2014). The wide-ranging effects of these molecules underlines the crucial need to fully annotate and study sRNAs in individual organisms, from both a functional and an evolutionary perspective.

The versatile genus *Staphylococcus* encompasses a diverse set of organisms that range from highly pathogenic to food-grade species. Staphylococci live on the mucous membranes of virtually all animals, as well as in aged meat products. *Staphylococcus carnosus* is an avirulent, coagulase-negative member of the staphylococci with the highest G+C content, and is commonly used as a starter culture for fermented sausages (Rosenstein *et al.*, 2009; Schleifer & Fischer, 1982; Wagner *et al.*, 1998). The genome of *S. carnosus*, an organism often regarded as an ancient and genetically simple species, generally has a lack of mobile genetic elements, especially in comparison with the other staphylococci. In contrast, *Staphylococcus epidermidis* is a coagulase-negative, opportunistic pathogen that is found as a part of the normal human flora of the skin and nares (Otto, 2009). *S. epidermidis* infections often occur through indwelling devices such as catheters, but are rarely life threatening or invasive (Otto, 2009). *Staphylococcus aureus*, also a normal part of the human flora, is a coagulase-positive member of the staphylococci, and is one of the leading causes of human infectious disease and death. *S. aureus* causes a wide variety of infections, ranging from minor cellulitis to life-threatening sepsis, and is capable of infecting all organ systems (Archer, 1998; Lowy, 1998). Compounding its extensive pathogenicity is the widespread prevalence of antibiotic-resistant isolates, which severely limits the number of viable treatment options (Lowy, 2003).

**Fig. 1.**
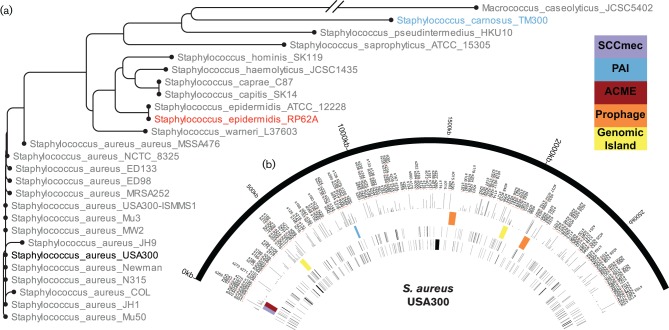
Staphylococcal phylogeny and sRNA content. (a) Phylogenetic relationship was determined using the *rpoB* gene from a range of *S. aureus* isolates, alongside other species within the *Staphylococcaceae*. The three strains from this study (*S. carnosus* TM300, *S. epidermidis* RP62A and *S. aureus* USA300) are highlighted (blue, red and black, respectively) within the tree. The tree was created using the CLC Main Workbench software and default settings. The *rpoB* gene sequences were retrieved from NCBI. (b) Circos file representing the *S. aureus* USA300 genome with recently annotated sRNAs (Carroll *et al.*, 2016). Depicted from the outermost semicircle inward are: the genome of *S. aureus*, sRNA annotations, expression level of each sRNA under standard conditions, genomic landmarks [SCCmec (purple), pathogenicity islands (red), prophages (orange) and other genomic islands (yellow)], sRNAs encoded on the forward strand and, innermost, the reverse strand.

Collectively, the diversity of lifestyles and evolutionary relationships between the staphylococci (Fig. 1) make this a model genus to ask how regulatory molecules change and adapt across species; and how they develop specialized, and niche-specific functions within a given organism. As such, in this study we identified and annotated the sRNA content of both *S. epidermidis* and *S. carnosus* using next-generation sequencing technologies coupled with comparative genomics. These newly annotated sRNAs were analysed for homology to each other, and to those recently curated by our group for *S. aureus* (Carroll *et al.*, 2016), to identify conserved and unique elements for each species. In total, we identified 118 total sRNAs in *S. epidermidis* and 89 in *S. carnosus,* compared with 303 in *S. aureus* (Carroll *et al.*, 2016). A comparison of these datasets revealed that each genome contains between 36 and 53 sRNAs that are common to all three organisms. Finally, we uncovered the presence of several highly homologous sRNAs in *S. epidermidis* and *S. aureus* that share conserved sequences, and appear to retain common structural motifs. Collectively, our work shines a light on these complex and largely overlooked regulators, providing insight into staphylococcal speciation, and the evolution of pathogenesis within this genus.

## Methods

### Bacterial strains and growth conditions.

*S. epidermidis* RP62a (Gill *et al.*, 2005) and *S. carnosus* TM300 (Schleifer & Fischer, 1982; Rosenstein *et al.*, 2009) were cultivated in tryptic soy broth (TSB), with shaking (250 r.p.m.) at 37 °C overnight. Synchronous cultures were achieved as outlined by us previously (Kolar *et al.*, 2011) before being grown for 3 h to the exponential phase.

### RNAseq.

Transcriptomic experiments using an Ion Torrent Personal Genome Machine (PGM) system (Ion Torrent) were performed as described by us previously (Carroll *et al.*, 2014). Briefly, total RNA was isolated from exponentially growing cultures using an RNeasy kit (Qiagen), with DNA removed using a TURBO DNA-free kit (Ambion). Next, RNA integrity was confirmed utilizing an Agilent 2100 Bioanalyzer system in combination with a RNA 6000 Nano Kit (Agilent). To remove rRNA from samples, a Ribo-Zero rRNA Removal Kit (Bacteria) (Epicentre) and MICROBExpress Bacterial mRNA Enrichment Kit (Ambion) were used in a sequential approach; complete removal of rRNA species was confirmed using an Agilent RNA 6000 Nano Kit. cDNA libraries were constructed from the enriched RNA with an Ion Total RNA-seq Kit v2 (Ion Torrent), before cDNA fragments were amplified onto Ion Sphere Particles (ISPs) using an Ion PGM Template OT2 200 Kit (Ion Torrent) and an Ion OneTouch 2 system (Ion Torrent). Template-positive ISPs were subsequently loaded onto Ion 318 v2 chips (Ion Torrent) and sequencing runs were performed utilizing an Ion PGM Sequencing 200 Kit v2 (Ion Torrent). After completion of each run, data were imported to the CLC Genomics Workbench software (CLC bio; Qiagen) and aligned to the publicly available *S. epidermidis* RP62a (NCBI accession number: NC_002976.3) and *S. carnosus* TM300 (NCBI accession number: AM295250.1) genomes. The addition of novel annotations to the *S. epidermidis* and *S. carnosus* genomes was performed according to guidelines outlined by us previously (Carroll *et al.*, 2016; Weiss *et al*., 2015). Updated annotation files including novel sRNA transcripts for *S. epidermidis* RP62a and *S. carnosus* TM300 were deposited to Figshare (Data citation 1). The annotation files containing sRNA annotations were used to generate expression values calculated as RPKM (reads per kilobase material per million reads) in CLC Genomics Workbench. All downstream bioinformatic analyses (e.g. blast searches investigating sRNA similarities between different species) were also performed with CLC Genomics Workbench software. RNA structure predictions were performed using the mfold web server (Zuker, 2003).

### Northern blots.

To confirm the presence of novel transcripts identified by RNAseq, we performed Northern blot analysis for selected sRNA candidates. Northern blots were performed as outlined previously (Caswell *et al.*, 2012), as follows. RNA from exponentially growing cultures was isolated and DNA-depleted as described for RNAseq samples. RNA was electrophoretically separated in a 10 % polyacrylamide gel [1x× TBE (Tris/borate/EDTA) buffer, 7 m urea] and transferred to an Amersham Hybond N+ membrane (GE Healthcare) by electroblotting. Samples were crosslinked to membranes via UV radiation, followed by pre-hybridization in ULTRAhyb-Oligo buffer (Ambion) for 1 h at 43 °C in a rotating oven. Next, [γ-^32^P]-ATP end-labelled oligonucleotides specific for each target RNA sequence (Table S1, available in the online Supplementary Material) were added to membranes and hybridized overnight at 43 °C. The following day membranes were washed with 2×, 1× and 0.5× SSC (saline and sodium citrate) buffer for 30 min at 43 °C. Finally, membranes were exposed to X-ray film to detect radiolabelled and specifically bound probes.

## Results

### Annotation of sRNAs in the *S. epidermidis* RP62a and *S. carnosus* TM300 genomes

The goal of this study was to gain insight into the impact of sRNAs on staphylococcal species-specific adaptation. A set of organisms was chosen to represent the diverse lifestyles of staphylococcal species: *S. aureus* USA300-Houston, an epidemic community-associated methicillin-resistant strain isolated from the wrist abscess of a 36-year-old, HIV-positive, intravenous drug user; *S. epidermidis* RP62a, a methicillin-resistant strain isolated from a patient suffering from intravascular catheter-associated sepsis; and *S. carnosus* TM300, originally isolated from dry sausage in 1982 in Germany (Highlander *et al.*, 2007; Gill *et al.*, 2005; Schleifer & Fischer, 1982; Rosenstein *et al.*, 2009). Importantly, these organisms are intermediately and distanty related species (Fig. 1a), representing the highly virulent (*S. aureus*), the mildly virulent (*S. epidermidis*) and the avirulent (*S. carnosus*). As such, they have the potential to provide significant insight into those sRNAs that are core to the staphylococci, as well as those that influence species-specific adaptation. Recently we re-annotated the genome of *S. aureus* (Carroll *et al.*, 2016) to include all sRNAs from the literature, as well as several novel transcripts identified by our group using next-generation sequencing approaches (Fig. 1b). As such, we used a similar RNAseq-based approach to re-annotate the genomes of *S. epidermidis* RP62a and *S. carnosus* TM300.

To our knowledge, no sRNAs have been identified or studied in either *S. epidermidis* or *S. carnosus* to date, and neither published genome has any sRNAs currently annotated. Given the absence of any information regarding the sRNAs of these two species, a transcriptomic approach was used to identify sRNAs in these genomes. Initially, each RNAseq was performed on cultures grown to the mid-logarithmic phase, with all reads generated aligned to the published genomes of *S. epidermidis* RP62a and *S. carnosus* TM300 (Gill *et al.*, 2005; Schleifer & Fischer, 1982; Rosenstein *et al.*, 2009). Files were then reviewed for the presence of sRNA reads using criteria defined by us previously for *S. aureus* and *Acinetobacter* *baumannii* (Carroll *et al.*, 2016; Weiss *et al*., 2015), as: antisense to previously annotated protein coding genes (Fig. S1a), in intergenic regions (Fig. S1b) or that showed differential expression from annotated genes with which they overlapped (Fig. S1c).

### The first genome-wide identification of sRNAs in *S. epidermidis* and *S. carnosus*

**Fig. 2.**
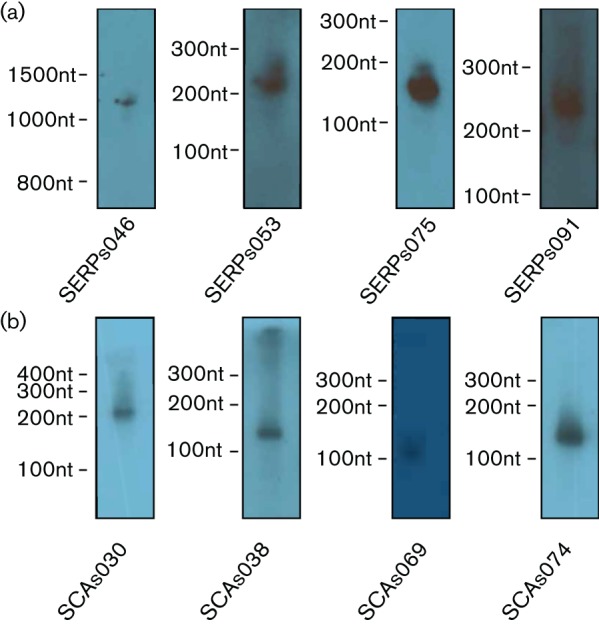
Northern blot analysis of sRNAs in *S. epidermidis* and *S. carnosus.* Total RNA was isolated from *S. epidermidis* RP62a (a) and *S. carnosus* TM300 (b) cultures grown to the mid-logarithmic phase. Samples were analysed using DNA probes specific to each transcript. Size markers, and the RNA probed for, are denoted on each gel.

In total, 118 and 89 sRNAs were identified in *S. epidermidis* RP62a and *S. carnosus* TM300, respectively (Tables 1 and 2). The sRNAs in each organism are distributed across their respective chromosomes, with the exception of a general lack of sRNAs in regions encoding prophages. The lack of sRNAs residing in these regions is perhaps to be expected, as these are relatively recent evolutionary events that have not yet been homogenized into the rest of the genome. To facilitate the addition of novel sRNA annotations in the future, an annotation system was created that does not relate to function, but instead acts only as an identifier (as described by us for *S. aureus*) (Carroll *et al.*, 2016). As such, sRNAs from *S. epidermidis* were denoted as SERPs001–SERPs118, referring to their total number, for ease of sequential incorporation of new sRNA annotations in the genome. Similarly, in *S. carnosus* sRNAs were denoted as SCAs001–SCAs089. Newly annotated genes were given the gene names *jointly annotated epidermidis loci* (*jaeL*)1–118 and *jointly annotated carnosus loci* (*jacL*) 1–89 for *S. epidermidis* and *S. carnosus*, respectively. To confirm the size and expression of sRNAs discovered in *S. epidermidis* and *S. carnosus*, several representative transcripts were chosen for Northern blot validation (Fig. 2). Each of the sRNAs analysed produced a single, probe-specific band at the size suggested by RNAseq, and as annotated herein. These findings suggest that the methods used by our group to identify and annotate novel sRNAs are both robust and reproducible.

**Table 1.** Newly annotated non-coding RNAs of *S epidermidis* RP62a

Locus ID	Gene name	Position	Upstream*	Orientation	Downstream*	RPKM†
SERPs001	*jaeL-1*	34999..35245	SERPs117	<	SERP0038	247.23
SERPs002	*jaeL-2*	42907..43025	SERP0050	<	SERP0051	125.96
SERPs003	*jaeL-3*	953853..954050	SERPs113	>	*trpE*	11.22
SERPs004	*jaeL-4*	60337..60509	*guaA*	<	SERP0071	327.32
SERPs005	*jaeL-5*	586811..586861	SERPs022	<	SERP0593	21.77
SERPs006	*jaeL-6*	183818..183969	*rplA*	>	*rplJ*	2081.83
SERPs007	*jaeL-7*	212119..212293	SERP0201	<	SERP0202	367.99
SERPs008	*jaeL-8*	233449..233600	SERP0223	<	SERP0224	1281.97
SERPs009	*jaeL-9*	233917..234080	SERP0224	>	SERP0225	67.7
SERPs010	*jaeL-10*	241408..241559	SERP0234	>	SERP0235	927.69
SERPs011	*jaeL-11*	307532..307673	SERP0304	<	SERP0305	328.4
SERPs012	*jaeL-12*	370776..370941	SERP0373	<	*pabA*	153.84
SERPs013	*jaeL-13*	377942..378056	SERP0379	<	SERP0380	77.24
SERPs014	*jaeL-14*	393987..394130	SERP0391	>	SERP0392	34.7
SERPs015	*jaeL-15*	443311..443466	SERP0438	>	SERP0439	1633.44
SERPs016	*jaeL-16*	469574..469690	SERP0466	<	SERP0467	469.75
SERPs017	*jaeL-17*	475770..475890	SERP0477	<	SERP0478	50.47
SERPs018	*jaeL-18*	480754..480892	SERP0488	>	SERP0489	7045.28
SERPs019	*jaeL-19*	526358..526540	SERP0542	<	SERP0543	54.61
SERPs020	*jaeL-20*	570220..570273	*trpS*	>	SERP0576	452.35
SERPs021	*jaeL-21*	576526..576580	SERPs082	>	SERP0581	2967.56
SERPs022	*jaeL-22*	586669..586828	SERP0592	>	SERPs121	190.83
SERPs023	*jaeL-23*	592434..592529	SERP0599	<	SERP0600	763.34
SERPs024	*jaeL-24*	658364..658539	SERP0662	<	SERP0663	466.83
SERPs025	*jaeL-25*	695170..695337	SERP0697	<	SERP0698	168.53
SERPs026	*jaeL-26*	717301..717513	SERP0720	>	*pheS*	297.13
SERPs027	*jaeL-27*	817973..818158	*sucD*	<	SERP0815	984.95
SERPs028	*jaeL-28*	964074..964190	*femB*	<	SERP0948	332.14
SERPs029	*jaeL-29*	981290..981497	SERP0962	>	*lysC*	10366.45
SERPs030	*jaeL-30*	1007973..1008125	SERP0990	>	SERP0991	486.21
SERPs031	*jaeL-31*	1099406..1099581	SERP1053	<	*srrB*	1526.68
SERPs032	*jaeL-32*	1150188..1150367	SERP1109	>	SERP1110	77.1
SERPs033	*jaeL-33*	1227591..1227843	SERP1191	<	*aspS*	4785.74
SERPs034	*jaeL-34*	1231240..1231434	*hisS*	<	SERP1194	1773.65
SERPs035	*jaeL-35*	1279465..1279597	*infC*	<	SERP1245	346.45
SERPs036	*jaeL-36*	1281335..1281543	SERP1245	<	*thrS*	1500.78
SERPs037	*jaeL-37*	1308770..1308884	*dnaE*	<	SERP1267	1042.73
SERPs038	*jaeL-38*	1318633..1318805	SERP1273	<	SERP1274	67.39
SERPs039	*jaeL-39*	1337905..1338103	SERP1292	>	*tyrS*	544
SERPs040	*jaeL-40*	1379890..1380079	*leuS*	<	SERP1319	978.83
SERPs041	*jaeL-41*	1389878..1390104	*ribD*	<	SERP1329	2298.88
SERPs042	*jaeL-42*	1409579..1409745	*metK*	<	*pckA*	1618.92
SERPs043	*jaeL-43*	1518292..1518416	*pheA*	<	SERP1453	31.09
SERPs044	*jaeL-44*	1542695..1542748	SERP1479	>	SERP1480	318.7
SERPs045	*jaeL-45‡*	1557626..1557897	SERP1488	<	*hld*	289.82
SERPs046	*jaeL-46*	1647845..1649295	SERP1623	>	SERP1624	3008.01
SERPs047	*jaeL-47*	292616..292825	SERPs119	<	*sitC*	795.72
SERPs048	*jaeL-48*	1744046..1744193	SERP1701	<	*sceD*	213.81
SERPs049	*jaeL-49*	1769544..1769767	*murAB*	<	*fbaA*	2614.68
SERPs050	*jaeL-50*	1774668..1774750	*pyrG*	<	*rpoE*	3083.45
SERPs051	*jaeL-51*	1790875..1791019	SERP1753	<	SERP1754	298.64
SERPs052	*jaeL-52*	292457..292605	SERP0289	<	SERPs120	1538.79
SERPs053	*jaeL-53*	1905371..1905605	SERP1880	<	*nhaC*	1946.59
SERPs054	*jaeL-54*	1917116..1917271	SERP1894	<	SERP1895	327.4
SERPs055	*jaeL-55*	1935344..1935518	SERP1914	<	SERP1915	396.54
SERPs056	*jaeL-56*	1997129..1997489	*sarZ*	>	SERP1980	522.86
SERPs057	*jaeL-57*	2014397..2014589	SERP1994	<	SERP1995	175.46
SERPs058	*jaeL-58*	2096196..2096389	SERP2069	>	SERP2070	354.84
SERPs059	*jaeL-59*	2110878..2111312	SERP2083	<	*aldA-2*	98.27
SERPs060	*jaeL-60*	2118226..2118357	SERP2091	<	SERP2092	786.47
SERPs061	*jaeL-61*	2184407..2184483	*ldh*	<	SERP2157	439.8
SERPs062	*jaeL-62*	2206812..2206918	SERP2175	<	*betA*	15.57
SERPs063	*jaeL-63*	2251118..2251304	SERP2212	>	SERPs064	724.37
SERPs064	*jaeL-64*	2251300..2251353	SERPs063	<	SERP2213	586
SERPs065	*jaeL-65*	2258818..2259071	*cadC*	>	SERPs066	657.88
SERPs066	*jaeL-66*	2259072..2259128	SERPs065	<	SERP2223	2084.27
SERPs067	*jaeL-67*	2266286..2266340	SERP2235	<	SERP2236	403.75
SERPs068	*jaeL-68*	2301741..2301875	SERP2268	>	SERPs069	16.45
SERPs069	*jaeL-69*	2301905..2301964	SERPs068	<	SERP2269	120.28
SERPs070	*jaeL-70*	2352134..2352256	*mqo-3*	<	SERP2313	216.65
SERPs071	*jaeL-71*	2400262..2400406	SERP2353	<	SERP2354	72.74
SERPs072	*jaeL-72*	2508708..2508954	SERP2454	<	SERP2455	384.34
SERPs073	*jaeL-73*	2519862..2520195	SERP2465	<	SERP2466	28.26
SERPs074	*jaeL-74*	2550198..2550302	*kdeP*	<	SERP2491	158.62
SERPs075	*jaeL-75*	2574243..2574391	SERP2518	>	*mecI*	21766.54
SERPs076	*jaeL-76*	2600571..2600779	SERP2541	<	SERP2542	159.37
SERPs077	*jaeL-77*	2605191..2605352	SERP2546	<	SERP2547	376.96
SERPs078	*jaeL-78*	297416..298093	*tagA*	<	SERP0296	133.47
SERPs079	*jaeL-79*	484345..485243	SERP0494	>	SERP0495	397.69
SERPs080	*jaeL-80*	1799010..1799177	SERP1761	<	*glmM*	218.1
SERPs081	*jaeL-81*	1848280..1848453	SERP1803	<	*rplQ*	618.97
SERPs082	*jaeL-82*	576366..576526	*pepF*	>	SERPs021	303.44
SERPs083	*jaeL-83*	1702694..1702924	SERP1664	>	*ilvD*	4.81
SERPs084	*jaeL-84*	1862469..1862606	*rpsJ*	<	SERP1833	1890.75
SERPs085	*jaeL-85*	1844698..1844868	*rplM*	<	*truA*	879.81
SERPs086	*jaeL-86*	755335..755547	SERP0757	>	*ileS*	364.89
SERPs087	*jaeL-87*	1283598..1283838	*thrS*	<	*dnaI*	377.78
SERPs088	*jaeL-88*	2475827..2476027	SERP2413	<	SERP2414	116
SERPs089	*jaeL-89*	1146822..1146987	*gcvT*	<	*aroK*	662.17
SERPs090	*jaeL-90*	185734..185859	SERP0182	>	*rpoB*	74.9
SERPs091	*jaeL-91*	603425..603663	*prfC*	>	SERP0610	308.94
SERPs092	*jaeL-92*	1406055..1406237	SERP1349	>	SERP1350	21.24
SERPs093	*jaeL-93*	1238590..1238761	*recJ*	<	SERP1200	22.59
SERPs094	*jaeL-94*	1449425..1449591	SERP1393	>	SERP1394	13.3
SERPs095	*jaeL-95*	2020634..2020835	*fmhA*	<	SERP2002	118.18
SERPs096	*jaeL-96*	632659..632761	SERP0638	>	SERP0639	21.56
SERPs097	*jaeL-97*	1932904..1933097	SERP1912	>	SERP1913	57.23
SERPs098	*jaeL-98*	2173475..2173629	SERP2146	>	SERP2147	25.07
SERPs099	*jaeL-99*	550036..550252	SERP0558	<	SERP0559	28.14
SERPs100	*jaeL-100*	367959..368097	SERP0369	>	SERP0370	27.96
SERPs101	*jaeL-101*	950460..950575	SERP0933	>	*dmpI*	23.93
SERPs102	*jaeL-102*	1142437..1142535	SERP1099	>	SERPs104	33.65
SERPs103	*jaeL-103*	1444172..1444269	SERP1386	<	*fumC*	385.21
SERPs104	*jaeL-104*	1142550..1142729	SERPs102	<	SERP1100	259.07
SERPs105	*jaeL-105*	2192309..2192465	SERP2163	>	SERP2164	17.68
SERPs106	*jaeL-106*	2360082..2360248	SERP2323	<	SERP2324	9.97
SERPs107	*jaeL-107*	54838..54966	SERP0066	>	*xpt*	210.87
SERPs108	*jaeL-108*	617908..618225	SERP0626	>	*menA*	80.31
SERPs109	*jaeL-109*	485246..485535	SERP0495	>	*sufC*	45.94
SERPs110	*jaeL-110*	1263896..1264162	*valS*	<	SERP1229	380.5
SERPs111	*jaeL-111*	1339993..1340256	*tyrS*	<	SERP1294	172.43
SERPs112	*jaeL-112*	328604..328788	SERP0323	<	*scdA*	96.03
SERPs113	*jaeL-113*	953678..953822	*tyrA*	>	SERPs122	7.66
SERPs114	*jaeL-114*	176298..176556	*gltX*	>	*cysE*	182.19
SERPs115	*jaeL-115*	1217236..1217468	*alaS*	<	SERP1183	457.47
SERPs116	*jaeL-116*	1170711..1170867	SERP1131	>	*glyS*	495.04
SERPs117	*jaeL-117*	34833..34995	SERP0037	<	SERPs001	136.23
SERPs118	*jaeL-118*	847682..847797	*ribF*	>	*rpsO*	641.3

*Gene.

†RPKM, reads per kilobase material per million reads.

‡Region corresponds to portion of RNAIII.

**Table 2.** Newly annotated non-coding RNAs of *S. carnosus* TM300

Locus ID	Gene name	Position	Upstream*	Orientation	Downstream*	RPKM†
SCAs001	*jacL-1*	9628..9890	SCA_0010	<	SCA_0011	329.72
SCAs002	*jacL-2*	16355..16590	SCAs079	<	SCA_0015	489.92
SCAs003	*jacL-3*	68516..68644	*fcbC*	>	SCA_0067	1331.56
SCAs004	*jacL-4*	114686..114974	SCA_0114	>	SCA_0115	1318.64
SCAs005	*jacL-5*	144461..144729	SCA_0141	>	SCAs006	212.44
SCAs006	*jacL-6*	144466..144711	SCAs005	<	SCA_0142	4.05
SCAs007	*jacL-7*	195809..196025	*gltX*	>	*cysE*	736.45
SCAs008	*jacL-8*	239896..240240	*proP*	<	*thiD*	1253.87
SCAs009	*jacL-9*	248359..248731	SCA_0240	>	SCA_0241	26355.27
SCAs010	*jacL-10*	286557..286798	*tagA*	>	*thrS*	6.86
SCAs011	*jacL-11*	302807..302992	SCA_0293	<	SCA_0294	7754.21
SCAs012	*jacL-12*	349042..349212	*norA*	>	SCA_0342	443
SCAs013	*jacL-13*	370812..371006	SCA_0365	>	SCA_0366	386.77
SCAs014	*jacL-14*	386729..386902	SCA_0377	>	SCAs090	324.61
SCAs015	*jacL-15*	409625..409751	SCA_0399	>	SCA_0400	7319.89
SCAs016	*jacL-16*	435453..435577	SCA_0420	>	SCA_0421	459.83
SCAs017	*jacL-17*	439576..439686	*gapA*	>	*pgk*	12059.66
SCAs018	*jacL-18*	449553..449930	*smpB*	>	NEW_REGION_507	40323.14
SCAs019	*jacL-19*	465254..465398	SCA_0454	>	SCA_0455	6846.57
SCAs020	*jacL-20*	475448..475663	*int*	>	SCA_0464	189.2
SCAs021	*jacL-21*	478847..479060	SCA_0469	<	SCA_0470	172.33
SCAs022	*jacL-22*	519236..519323	*glpQ*	>	SCA_0522	211.43
SCAs023	*jacL-23*	704781..704974	SCA_0700	<	SCA_0701	438.43
SCAs024	*jacL-24*	736699..736810	SCA_0733	<	SCA_0734	3740.74
SCAs025	*jacL-25*	754215..755140	SCA_0752	>	SCA_0753	236.45
SCAs026	*jacL-26*	809208..809442	*rluD*	>	*pyrR*	10723.79
SCAs027	*jacL-27*	954424..954574	*mutS*	>	*mutL*	28.6
SCAs028	*jacL-28*	1026107..1026242	SCA_1009	<	*tyrA*	2460.09
SCAs029	*jacL-29*	1028624..1028907	SCA_1011	>	*trpE*	926.55
SCAs030	*jacL-30*	1055393..1055592	SCA_1036	>	*lysC*	3098.2
SCAs031	*jacL-31*	1080768..1080936	SCA_1060	>	SCA_1061	139.58
SCAs032	*jacL-32*	1098995..1099432	SCA_1079	<	SCA_1080	1725.71
SCAs033	*jacL-33*	1103951..1104094	*pbp2*	<	SCA_1085	230.73
SCAs034	*jacL-34*	1136220..1136427	SCA_1116	<	*srrB*	1380.1
SCAs035	*jacL-35*	1239320..1239485	*alaS*	<	SCA_1231	28.02
SCAs036	*jacL-36*	1249565..1249804	SCA_1240	<	*asp*S	5994.28
SCAs037	*jacL-37*	1252944..1253215	*hisS*	<	SCA_1243	2385.58
SCAs038	*jacL-38*	1294117..1294234	*infC*	<	*lysP*	23251.64
SCAs039	*jacL-39*	1339350..1339494	SCA_1325	>	*rpsD*	1271.7
SCAs040	*jacL-40*	1354036..1354126	*fhs*	<	*acsA*	200.81
SCAs041	*jacL-41*	1373449..1373678	SCA_1356	<	*dat*	1246.65
SCAs042	*jacL-42*	1383627..1383700	*leuS'*	>	SCA_1366	9195.15
SCAs043	*jacL-43*	1393914..1394102	*ribD*	<	SCA_1376	1063.54
SCAs044	*jacL-44*	1406587..1406766	*metK*	<	*pckA*	2198.37
SCAs045	*jacL-45*	273470..273659	*sarA*	<	SCA_0267	34.97
SCAs046	*jacL-46*	1546771..1546977	SCA_1526	<	SCA_1527	500.78
SCAs047	*jacL-47*	1562210..1562443	SCA_1544	>	*agrB*	985.38
SCAs048	*jacL-48*	1585607..1585749	*ilvA*	<	23S rRNA D	2012.07
SCAs049	*jacL-49*	1664220..1664368	SCA_1648	>	SCA_1649	196.23
SCAs050	*jacL-50*	1824275..1824411	SCA_1822	<	SCA_1823	637.82
SCAs051	*jacL-51*	1917867..1918006	SCA_1912	<	SCA_1913	832.99
SCAs052	*jacL-52*	1929303..1929527	SCA_1921	<	SCA_1922	84.17
SCAs053	*jacL-53*	1941508..1941755	*opuCA*	<	SCA_1935	515.79
SCAs054	*jacL-54*	2031863..2032724	SCA_2018	>	SCA_2019	7319.06
SCAs055	*jacL-55*	2088262..2088525	SCA_2075	>	SCA_2076	761.4
SCAs056	*jacL-56*	2199217..2199338	SCA_2172	<	SCA_2173	1628.55
SCAs057	*jacL-57*	2224729..2224907	SCA_2191	<	SCA_2192	848.25
SCAs058	*jacL-58*	2266552..2266687	SCA_2211	>	SCAs059	4277.68
SCAs059	*jacL-59*	2266688..2266868	SCAs058	<	SCAs060	2180.71
SCAs060	*jacL-60*	2267000..2267107	SCAs059	>	SCAs061	1239.77
SCAs061	*jacL-61*	2267108..2267271	SCAs060	<	*tatA*	1847.62
SCAs062	*jacL-62*	2294415..2294594	SCA_2236	<	SCA_2237	265.8
SCAs063	*jacL-63*	2303997..2304203	SCA_2247	>	SCAs064	1295.28
SCAs064	*jacL-64*	2304288..2304494	SCAs063	>	SCA_2248	166.93
SCAs065	*jacL-65*	2312205..2312796	SCA_2253	<	SCAs066	2442.46
SCAs066	*jacL-66*	2313086..2313451	SCAs065	>	SCA_2254	2055.21
SCAs067	*jacL-67*	2322081..2322191	*acsA*	>	*putP*	1655.25
SCAs068	*jacL-68*	2375256..2375403	SCA_2309	>	SCAs069	60.61
SCAs069	*jacL-69*	2375475..2375602	SCAs068	>	SCAs070	3569.06
SCAs070	*jacL-70*	2375660..2375782	SCAs069	<	*opuD*	27.01
SCAs071	*jacL-71*	387163..387583	SCAs014	>	*nrdI*	153.1
SCAs072	*jacL-72*	2550964..2551426	SCA_2464	>	*serS*	45.21
SCAs073	*jacL-73*	1295825..1296074	SCA_1288	<	*thrS*	1306.39
SCAs074	*jacL-74*	1732302..1732452	SCA_1707	<	*rplQ*	4134.38
SCAs075	*jacL-75*	756990..757182	SCA_0755	>	*pheS*	165.26
SCAs076	*jacL-76*	1639798..1639959	*rpmE*	<	*rho*	514.78
SCAs077	*jacL-77*	2552910..2553191	*serS*	<	*hutH*	153.16
SCAs078	*jacL-78*	1574476..1574699	SCA_1558	>	*ilvD*	467.22
SCAs079	*jacL-79*	16200..16350	*metC*	<	SCAs002	699.7
SCAs080	*jacL-80*	203095..203214	*rplA*	>	*rplJ*	2129.15
SCAs081	*jacL-81*	1298095..1298341	*thrS*	<	*dnaI*	793.63
SCAs082	*jacL-82*	1728725..1728877	*rplM*	<	*truA*	297.5
SCAs083	*jacL-83*	1200679..1200816	SCA_1186	>	*glyS*	334.65
SCAs084	*jacL-84*	1674226..1674513	*glmS*	<	*mtlA*	57.68
SCAs085	*jacL-85*	365862..366036	SCA_0360	<	SCA_0361	32.28
SCAs086	*jacL-86*	1648746..1648822	*pyrG*	<	SCA_1631	207.11
SCAs087	*jacL-87*	1278047..1278317	*valS*	<	*tag*	202.29
SCAs088	*jacL-88*	605369..605416	*pepF*	>	SCA_0600	699.1
**SCAs089**	*jacL-89*	1811579..1811734	SCA_1806	<	SCA_1807	10.65

*Gene.

†RPKM, reads per kilobase material per million reads.

### Defining the core staphylococcal sRNA content

**Fig. 3.**
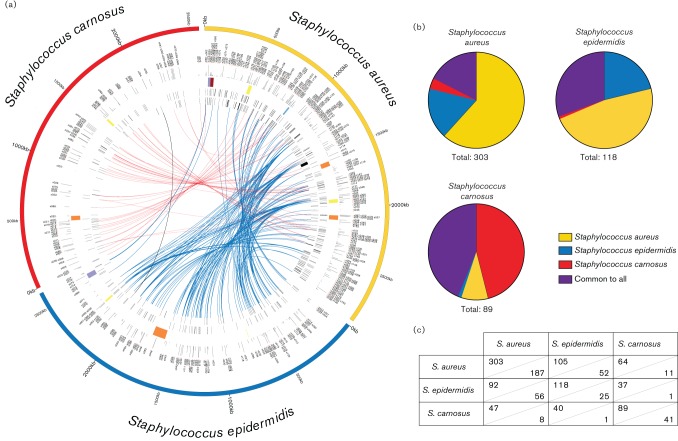
Shared and unique sRNA content amongst the staphylococci. (a) Depicted from the outermost semicircle inward are: the genome of *S. aureus*, sRNA annotations, expression level of each sRNA under standard conditions, genomic landmarks [SCCmec (purple), pathogenicity islands (red), prophages (orange) and other genomic islands (yellow)], sRNAs encoded on the forward strand and, innermost, the reverse strand. The inner links connect sRNAs that have sequence conservation. Red and blue links show homologous sRNAs between *S. aureus* and either *S. carnosus* or *S. epidermidis*, respectively; and the black link indicates a single homologous sRNA shared between *S. epidermidis* and *S. carnosus* but with no relation to any in *S. aureus.* (b) Pie charts representing the portion of sRNA content that is shared with each of the species in this study. The total sRNA content of each genome is indicated. (c) Numbers used to generate images in (b). Shown is the number of sRNAs shared between a given species pairing (upper section of each cell) as well as the number of sRNAs unique to a given species pairing (lower section of cells). For example, *S. aureus* has 105 sRNAs in common with *S. epidermidis*, but only 52 of these 105 are unique to *S. aureus* and *S. epidermidis* (i.e. not found in *S. carnosus*). When viewing these data, an organism-specific point-of-view must be employed to understand the differences in numbers from similar comparisons. Specifically, the numbers are different for *S. aureus* vs. *S. epidermidis* (105 and 52 sRNAs shared and specific, respectively) compared with *S. epidermidis* vs.* S. aureus* (92 and 56 sRNAs shared and specific, respectively) because *S. aureus* has 52 sRNAs that are homologous to 56 sRNAs in *S. epidermidis.*

Given that a primary goal of this study was to better understand the sRNAs that are specific to each species, and that may contribute to their individual lifestyles, we first set out to elucidate the shared sRNA content of the staphylococci (Fig. 3 and Table S2). An sRNA in one genome was considered homologous to another gene if blast searches returned an E-value ≤10^−10^ in a region that had been annotated. As such, we queried all sRNAs from each organism in a nucleotide blast search against the genomes of the other two staphylococcal species to gain a comprehensive overview of the shared and unique sRNAs encoded by each genome.

A confounding issue to this approach, however, is that there does not appear to be a 1 : 1 ratio of sRNAs from one organism to another. For example, a number of sRNAs from *S. aureus* have significant sequence homology to several sRNAs from *S. epidermidis* (described in more detailed below ). Indeed, this is not a lone occurrence as each organism comparison results in several such relationships. Accordingly, the unique and shared sRNA content of the staphylococci can only be specifically calculated from one genome to another, rather than across the genus as a whole. Such analyses are visually represented in Fig. 3(a) where links represent a homologous relationship between sRNAs of *S. aureus* and *S. epidermidis* (blue) or *S. carnosus* (red). A single sRNA exists in *S. epidermidis* and *S. carnosus* that shares homology to each other but has no relationship to any in *S. aureus* (black link). The relative (Fig. 3b) and absolute (Fig. 3c) number of shared sRNAs by genome vary significantly. At first glance it is readily apparent that nearly two-thirds of the sRNAs (187 of 303) previously identified in *S. aureus* are unique to this organism (Fig. 3b, c). *S. carnosus* has the next highest number of unique sRNAs, 41 of 89 (Fig. 3b, c). The high percentage of unique sRNAs in *S. carnosus* (~46 %) is perhaps to be expected, as it is the most distantly related of the three organisms in this study. In contrast, *S. epidermidis* has the least number of unique sRNAs, at 25 of 118 (~21 %) (Fig. 3c), meaning that nearly 79 % of its sRNA content is shared with *S. aureus* and/or *S. carnosus* (Fig. 3b, c). Collectively, we identified 53 core and 187 unique sRNAs in *S. aureus*, 36 core and 25 unique sRNAs in *S. epidermidis* and 39 core and 41 unique sRNAs in *S. carnosus* (Fig. 3b, c). The conservation of sequence and expression suggests that these sRNAs may be involved in more central and conserved processes, such as metabolism. As such, unique sRNAs may represent elements that are probably involved in individual, species-specific adaptation, which, in the case of *S. aureus*, suggests virulence processes.

**Fig. 4.**
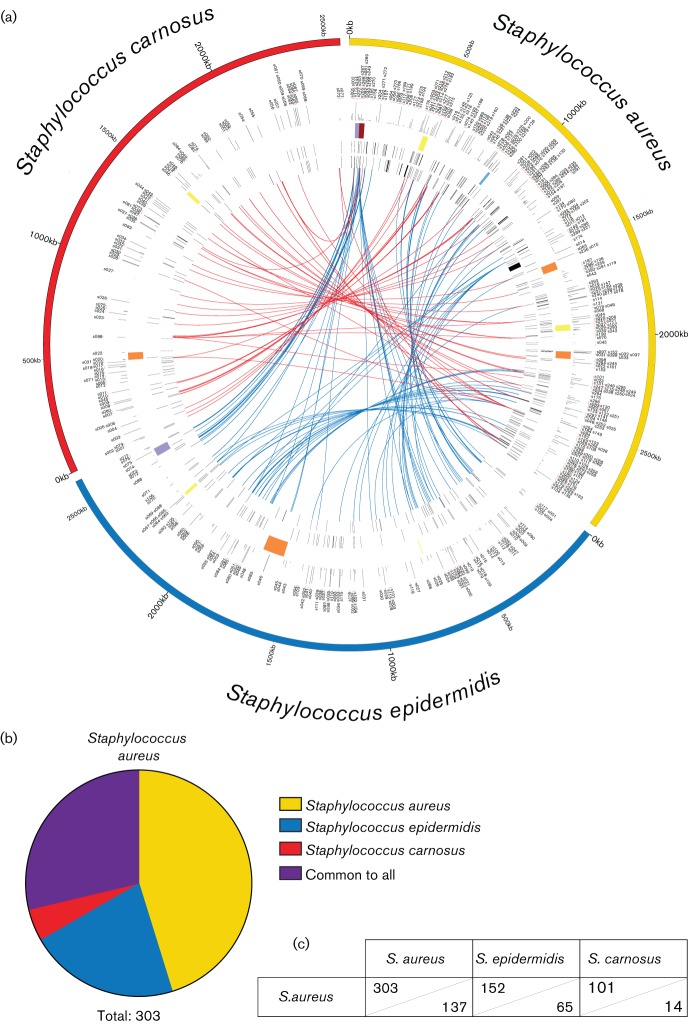
The identification of transcriptionally silent *S. aureus* sRNAs in *S. epidermidis* and *S.* *carnosus.* (a) Data are arranged in the same manner as Fig. 3(a), with the following differences: links connect annotated sRNAs of *S. aureus* to homologous regions within the chromosomes of *S. epidermidis* (blue) and *S. carnosus* (red) that do not show transcriptional activity in these latter species. Regions were considered homologous if blast search returned an E-value of <10^–10^. (b) Pie chart showing the total shared and unique sRNA content of *S. aureus* including the expressed sRNAs from Fig. 3 and the homologous unexpressed from (a). (c) Numbers used to generate images in (b). Shown are the number of small RNAs shared between a given species pairing (upper section of cells) as well as the number of sRNAs unique to a given species pairing (lower section of cells).

**Fig. 5.**
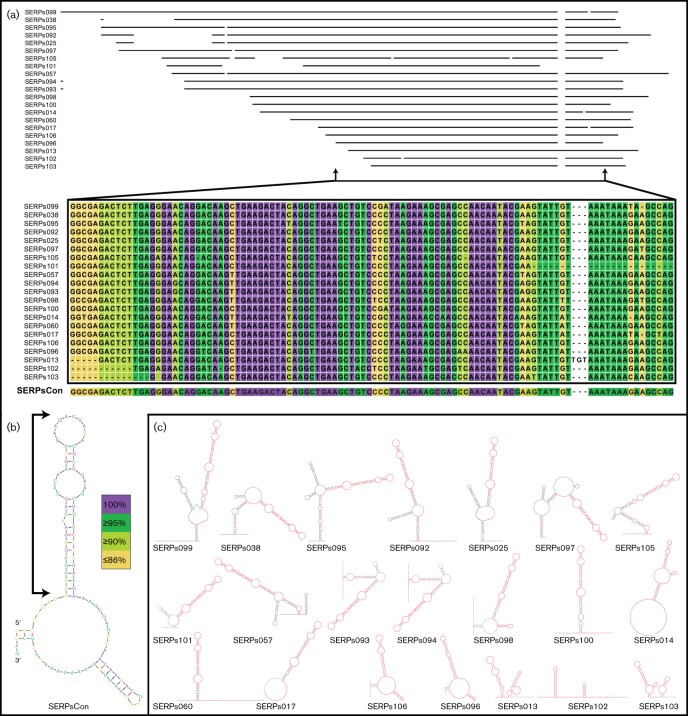
Sequence and structural conservation of several highly related and newly identified *S. epidermidis* sRNAs. (a) A sequence alignment of 21 newly identified sRNA genes from *S.* epidermidis, with a particular focus on the most conserved region within each. Within the zoomed in region, conservation at the nucleotide level is shown, with a consensus sequence generated from the alignment presented (SERPsCon). The level of conservation of each nucleotide is indicated by colour, and the number of sequences containing the conserved residue from all 21 sRNAs. Purple, conservation in all 21 sequences; dark green, 20/21; yellow, 19/21; orange, ≤ 18/21; and white, not conserved, and not included in the consensus sequence. Alignment, conservation analysis and consensus sequence generation were performed using the CLC Genomics Workbench software. (b) RNA secondary structure prediction for the consensus sequence generated in (a), with each residue colour coded to its level of conservation, as detailed in (a). RNA secondary structure predictions were generated using the mfold software. (c) RNA secondary structure predictions for each of the 21 sRNAs from the alignment. The most highly conserved region of each [from the zoomed in area in (a)] is highlighted in red. RNA secondary predictions were again generated using the mfold software.

A consideration with these data is that ours is the first study to evaluate *S. epidermidis* and *S. carnosus* sRNAs, which are derived from a single transcriptomic experiment. Conversely, studies by many groups, using a wealth of different approaches, have contributed to the 303 *S. aureus* sRNAs identified thus far. This is placed in context when one considers that the *S. aureus* sRNA content is greater than that from *S. epidermidis* (118 in total) and *S. carnosus* (89 in total) combined. As such, the possibility remains that several other sRNAs exist in these latter two species, but are not expressed under the conditions tested in our study. Accordingly, all sRNAs from *S. aureus* that showed significant sequence homology (E-value ≤10^−10^) to regions in the *S. epidermidis* or *S. carnosus* chromosomes were identified and denoted (Fig. 4a, Table S2). These regions were not annotated as sRNAs in the newly generated genome annotations, but their locations have been recorded (Table S2). While these loci did not show any transcriptional activity in *S. epidermidis* or *S. carnosus* in our study, they do share high sequence homology to known sRNAs of *S. aureus*, and thus may be expressed under different conditions not examined within this study. These transcriptionally inactive regions are linked to their homologous sRNA in *S. aureus* using blue and red links (*S. epidermidis* and *S. carnosus*, respectively) as before (Fig. 4a). When one factors these homologous, transcriptionally inactive regions into the shared and unique calculations, a very different picture appears (Fig. 4b, c). Specifically, the number of shared sRNAs increases greatly, elevating the putative *S. aureus* core-sRNA content from 53 to 87, whilst at the same time decreasing the number of unique sRNAs from 187 to 137.

**Fig. 6.**
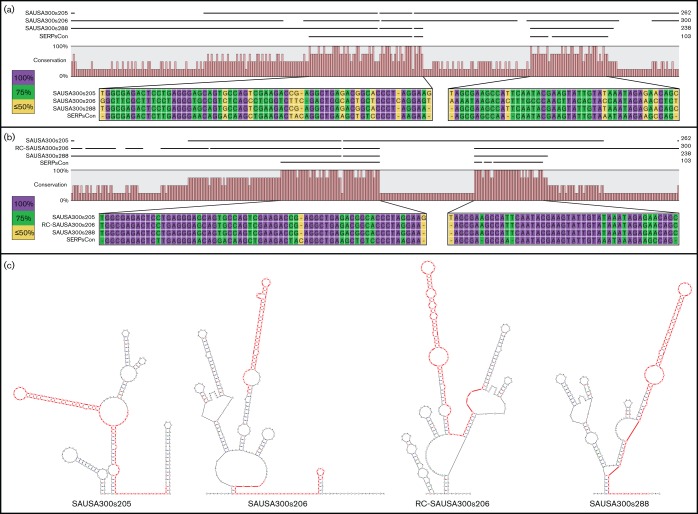
The *S. epidermidis* sequence and structural motif is conserved in homologous sRNAs in *S. aureus.* (a) Sequence alignment of three sRNA genes (SAUSA300s205, SAUSA300s206 and SAUSA300s288) from *S. aureus* and the consensus sequence generated (SERPsCon) in Fig. 4(a). Sequence annotations are shown on the left, and on the right total sequence length. Zoomed in areas show nucleotide conservation amongst the four sequences, with the conservation for each residue indicated by colour. Purple is 100 % conservation, green is 75 % and yellow is 50 % or below. (b) As in (a), but containing the reverse complement region of SAUSA300s206 (RC-SAUSA300s206) instead of its native orientation. (c) Secondary structure predictions for each *S. aureus* sRNA as well as RC-SAUSA300s206. Regions sharing a high level of homology to SERPsCon as determined in (a) (SAUSA300s206) or (b) (the rest) were highlighted in each structure prediction. RNA secondary predictions were generated using the mfold software.

### ORF prediction and conservation

The genomes of *S. aureus* USA300, *S. epidermidis* RP62a and *S. carnosus* TM300 have previously been annotated for standard genomic features, including origin of replication, tRNAs, rRNAs and protein-coding genes. During the automated annotation process, ORFs smaller than 50 codons in length are generally dismissed, but the importance of small peptides (those smaller than 50 aa) encoded by small ORFs is becoming increasingly recognized (Hobbs *et al.*, 2011; Storz *et al.*, 2014). As such, we examined the predicted ORF content of the newly annotated transcripts, as our annotation process does not exclude potential protein-coding genes (Tables S3 and S4). In *S. epidermidis*, only a single newly annotated transcript had a predicted ORF of 50 codons or longer, whilst 111 had predicted ORFs between five and 50 codons, and six had no predicted ORFs of five or more codons. Similarly, in *S. carnosus* six newly annotated transcripts had predicted ORFs greater than 50 codons, 73 had ORFs between five and 50 codons long and 11 sRNAs had no identifiable ORFs of five or more codons. Importantly, none of the predicted ORFs within each of the organisms examined had any significant homology to any protein with known function aside from the *S. aureus* Δ-hemolysin. Furthermore, the predicted ORFs from all three organisms also have very little similarity to each other, suggesting that these may not be translated (Tables S5 and S6). *S. epidermidis* and *S. carnosus* have a similar number of predicted ORFs per sRNA (3.3 ORFs and 3.9 ORFs per sequence, respectively) whereas the *S. aureus* sRNAs contain a much higher number of predicted ORFs (11.2 ORFs per sequence). This discrepancy is probably due to a difference in the average size of annotated sRNAs, as *S. aureus* has an average sRNA size of 506 nt compared with *S. epidermidis* and *S. carnosus* with 190 and 217 nt, respectively. As a note, the algorithm used to predict potential ORFs can predict more than one ORF per sRNA but does not evaluate the presence or absence of a ribosomal binding site. As such, the presence of an ORF does not provide any information on the likelihood of translation.

### An interspecies conserved and recurring sRNA structural motif

Initial investigations into the overall conservation of sRNA content in the staphylococci revealed the presence of a number of* S. epidermidis* elements with homology to sRNAs from *S. aureus (*Fig. 3a). Twenty-one sRNAs from *S. epidermidis* and three from *S. aureus* demonstrate a higher than random level of homology as first identified by blast analysis (Fig. 3a), and confirmed by sequence alignments (Figs 5a, 6a and S2). The sRNAs identified in *S. epidermidis* have a significantly higher level of nucleotide identity to each other (as determined by pairwise comparisons) than to the sRNAs of *S. aureus*, or that the *S. aureus* sRNAs do with each other (Figs S2–S4). Furthermore, while sequence conservation does exist between SAUSA300s206 and the other 23 sRNAs identified, it is the most divergent sequence (Figs 6a, S2 and S3).

The 21 highly related sRNA genes in *S. epidermidis* have a higher relative G+C content, ranging from 32.6 % for SERPs014 to 44.2 % for SERPs106, than the relative G+C content of the *S. epidermidis* genome (32.2 %). They also span a range of sizes from 98 bp (SERPs103) to 217 bp (SERPs099), with this variation seemingly attributable to differences in their 5' regions (Fig. 5a). Conversely, each sequence shares a key region of high conservation that extends approximately from the middle of the sequence to its 3′ end. This common region demonstrates nucleotide-level conservation of 71.4–100 %, not including the 3 bp insertion found in SERPs013. Using this information, we generated a consensus sequence (SERPsCon) that reflects the nucleotide identity of >71 % of the sequences in the conserved region.

The SERPsCon sequence and each *S. epidermidis* sRNA were subjected to secondary structure prediction using the mfold software (Fig. 5b, c, respectively) (Zuker, 2003). The predicted SERPsCon structure includes a stem with two single-stranded regions, and a terminal loop (bracketed) near the 5′ end of the molecule that includes 28 of the 38 residues conserved in all 21 sequences (Fig. 5a, b). The terminal single-stranded region of the SERPsCon structure within the bracket has a 10 nt sequence that is variable only at the ninth residue, and defined by the sequence motif 5′-GAAGACUAYA (Fig. 5b). Furthermore, mfold secondary structure predictions performed on the 21* S. epidermidis* sRNAs suggest the sequence homology extends to structural conservation. The secondary structure predictions suggest that in all 21 of these elements, the region corresponding to SERPsCon (Fig. 5c, red regions) includes an extended stem-loop structure that is identical (for 17 of the 21) to the motif defined in SERPsCon (5′-GAAGACUAYA). The remaining four sRNAs have the same sequence motif at the terminus of a stem, although the optimal structure, as predicted by mfold, suggests less single-strandedness. The conserved region and terminal loop do not appear to be related to any known RNA families or motifs as determined by an Rfam analysis (http://rfam.xfam.org/) (Nawrocki *et al.*, 2015), and thus may constitute a new regulatory RNA family.

Initial sequence analysis of SAUSA300s205, SAUSA300s206 and SAUSA300s288 determined that these sRNAs share less sequence similarity than their *S. epidermidis* counterparts (Figs S3 and S4). SAUSA300s205 and SAUSA300s288 are more similar to each other than to SAUSA300s206 (Figs 6a, S2 and S3), although upon further examination it was noted that SAUSA300s205 and SAUSA300s288 share more similarity with the reverse complement of SAUSA300s206 (RC-SAUSA300s206) (Figs 6b, S2 and S3). Secondary structure predictions suggest at least partial structural conservation between SAUSA300s205 and SAUSA300s288 in relation to SERPsCon and the other *S. epidermidis* sRNAs (Fig. 6c). The predicted secondary structure of SAUSA300s288 has the highest level of structure and sequence conservation, with nine residues in the terminal loop structure, seven of which are perfectly conserved in relation to SERPsCon (Fig. 5b). SAUSA300s205 contains a 59 nt insert within the region corresponding to SERPsCon that necessarily shifts the structure, resulting in a slightly lower level of sequence conservation in the terminal loop (six of nine residues) (Fig. 6c). Folding predictions of SAUSA300s206 suggest very little, if any, structural conservation, mirroring the lack of sequence similarity, with the other sRNAs (Fig. 6a, c). However, as perhaps is expected, mfold analysis of RC-SAUSA300s206 suggests structural conservation including the terminal loop (six of nine residues) (Fig. 6c).

The antisense nature of SAUSA300s206 in comparison with SAUSA300s205 and SAUSA300s288 hints at the possibility of an interaction between SAUSA300s206 and the other two sRNAs. To evaluate this potential, we queried the SAUSA300s206 sequence against the target sequences SAUSA300s205 and SAUSA300s288 using RNA–RNA interaction prediction software, IntaRNA (http://rna.informatik.uni-freiburg.de/) (Busch *et al.*, 2008; Wright *et al.*, 2014). Perhaps unsurprisingly, the predicted areas of interaction between SAUSA300s206 and both of the other sRNAs are extensive, and have a very low free energy (−181.7 and −95.3 kcal mol^−1^ for SAUSA300s205 and SAUSA300s288, respectively) thus making these interactions energetically favorable.

### Functional prediction of *S. aureus*-specific sRNAs

A major goal of this study was to differentiate sRNA content between the staphylococci (Table S2), and to garner a better understanding of the potential physiological role for unique elements, particularly in the context of *S. aureus* pathogenesis. As such, the complete set of *S. aureus*-specific sRNAs (Table S7) was subjected to target prediction using TargetRNA2 (http://cs.wellesley.edu/~btjaden/TargetRNA2/) (Kery *et al.*, 2014). The resulting list of putative targets (Table S8) was subjected to ontological classification, to identify those that are known virulence factors. Of note, 85 of the 137 (62 %) sRNAs unique to *S. aureus* were found to have the capacity to interact with at least one virulence-related transcript. Interestingly, the gene with the highest number of predicted sRNA regulators (10 different sRNAs) was *splA*, which encodes a well-characterized serine protease (Stec-Niemczyk *et al.*, 2009). This is particularly compelling as *S. aureus* proteases have a major role in pathogenesis via the global modulation of virulence determinant stability (Kolar *et al.*, 2013). As such, this clearly suggests potential for sRNA-based regulation of the infectious process in *S. aureus*. Ultimately, each of the predictions generated require further experimental verification to assess specific functional roles. However, we suggest that the data presented herein represent an important first step in exploring the influence of sRNAs in the staphylococci, and their impact on species-specific adaptation.

## Discussion

The advent of next-generation sequencing technologies has resulted in a vast amount of genomic and transcriptomic data available for all domains of life. This flood of data has resulted in the need for automated annotation software (Dark, 2013; Richardson & Watson, 2013). While automated annotation has become fairly robust for protein-coding regions, tRNAs and rRNAs, the ability to accurately predict the presence of other non-coding RNAs lags behind, which necessitates the manual curation of such genes (Sridhar & Gunasekaran, 2013). Collectively, sRNAs are of growing interest, as the diverse roles they play in regulating carbon metabolism, virulence gene expression, iron acquisition and many other cellular processes becomes increasingly apparent (Hoe *et al.*, 2013; Beisel & Storz, 2010; Murphy *et al.*, 2014; Caron *et al.*, 2010; Geissmann *et al.*, 2006; Harris *et al.*, 2013; Papenfort & Vanderpool, 2015; Oliva *et al.*, 2015). The inability to efficiently identify and annotate these elements hinders research on sRNAs and creates a need for transcriptomic-based approaches to supplement automated annotation software pipelines. To this end, our group has begun manually cataloguing and curating these molecules into their respective genomes within the staphylococci.

In the present work, we have identified and annotated sRNAs in the genomes of both *S. epidermidis* RP62a and *S. carnosus* TM300 using RNAseq methodologies. The total sRNA contents of *S. epidermidis* and *S. carnosus* were compared with our previous work in *S. aureus*, generating a fully comprehensive comparison of the shared and unique sRNA content of these common staphylococci. In so doing, we identified and annotated 118 and 89 novel sRNAs in *S. epidermidis* and *S. carnosus*, respectively. The sRNA content of these two genomes initially appears strikingly small compared with *S. aureus* (303 annotated sRNAs). The difference in the number of sRNAs between these organisms is probably not due to differences in genome size (2 872 769, 2 616 530 and 2 566 424 bp, respectively), but rather an artifact of the overall number of conditions tested for sRNA expression within each species. For *S. epidermidis* and *S. carnosus*, our study is the first assessment of their sRNA content, based on a single growth condition (mid-logarithmic phase, TSB at 37 °C), whereas those sRNAs for *S. aureus* are derived from a wealth of different studies and experimental conditions (Pichon & Felden, 2005; Marchais *et al.*, 2009; Geissmann *et al.*, 2009; Abu-Qatouseh *et al.*, 2010; Bohn *et al.*, 2010; Beaume *et al.*, 2010; Nielsen *et al.*, 2011; Xue *et al.*, 2014; Anderson *et al.*, 2006, 2010; Olson *et al*., 2011; Howden *et al.*, 2013; Carroll *et al.*, 2016). The discrepancy in the number of studies that have examined the sRNA content of these three organisms also underlies the very different proportion of sRNAs common to the staphylococci in each genome. For example, considering only the transcriptionally active sRNA comparisons, *S. aureus* has a common sRNA set of 53 (~17.5 %) while *S. epidermidis* and *S. carnosus* have 36 (~30.5 %) and 39 (~43.8 %), respectively. The sRNAs represented in all three genomes probably have similar roles within the cell, speculatively involved in evolutionary conserved processes such as basic metabolism and maintenance of cellular homeostasis. While the number of sRNAs shared by *S. aureus* increases to 87 (~28.7 %) if the homologous, but transcriptionally inactive, regions of *S. epidermidis* and* S. carnosus* are included, this is still a smaller proportion of the sRNAs compared with *S. epidermidis*, and considerably smaller than that of *S. carnosus* (~30.5 % and ~43.8 %, respectively). One could hypothesize that these sRNAs may be involved in conserved processes that are perhaps unnecessary under the conditions tested. Conversely, and of some interest, several regions within the *S. aureus* genome show high sequence similarity to newly annotated sRNAs from *S. epidermidis* and *S. carnosus*, despite themselves being transcriptionally silent (data not shown). Either the presence of such regions suggests an evolutionary event that has silenced expression from these loci, or, perhaps a more likely scenario, we have yet to elucidate the permissive conditions for their expression in *S. aureus.* As such, a need exists for further research into lifestyle-specific and patho-physiologically relevant transcriptomic conditions and effects within the staphylococci.

The presence of a set of highly conserved sRNAs from *S. epidermidis* and *S. aureus* is seemingly quite unusual. The high level of sequence similarity within these sRNAs also results in a conserved structural motif that takes the form of a stem and multi-loop region, ending in a terminal hairpin with an unpaired, conserved 9 – 10 nt motif. Conservation of the multi-loop stem and terminal loop would suggest a common function for these sRNAs as a group and/or for the region of homology. Several possibilities for general function present themselves with such sequence and structure conservation. For example, it is possible that these structures act to bind and sequester proteins, as is the case for the CsrB/C sRNAs. CsrB/C sRNAs were originally identified in *Escherichia coli* as binding to and sequestering the CsrA protein through a conserved, repeated RNA motif, ultimately affecting carbon utilization and virulence gene expression (Liu *et al*., 1997; Jonas & Melefors, 2009). A second scenario, that has been demonstrated for several of the Rsa sRNAs in *S. aureus* (first characterized for their UCCC motif), is that the terminal hairpin serves to bind conserved regions within a target RNA, and the surrounding, less conserved regions confer target specificity (Geissmann *et al*., 2009). More work is necessary to elucidate the function of each of these individual sRNAs as well as the conserved domain that characterizes them. Curiously, the homology searches also identified SAUSA300s206 within this group, although further *in silico* analysis demonstrated SAUSA300s206 has high sequence complementarity to SAUSA300s205 and SAUSA300s288 as well as SERPsCon. The presence of high levels of sequence complementarity begs the question: is SAUSA300s206 a regulator of SAUSA300s205 and SAUSA300s288? Regulation of one sRNA by another (so-called anti-sRNAs) is not unprecedented in the literature. In *E. coli* the molecular mechanism for interaction of two such anti-sRNAs, AsxR and AgvB, with their targets has recently been elucidated (Tree *et al.*, 2014). AsxR binds the sRNA FnrS, which normally represses the expression of a heme oxygenase, ChuS; thus, AsxR acts to enhance expression of ChuS (Tree *et al.*, 2014). In the context of AgvB, it binds the sRNA GcvB, repressing the GcvB-dependent repression of DppA expression (Tree *et al.*, 2014). In direct parallel to this, our group recently identified a set of highly transcribed, highly homologous sRNAs in *A. baumannii*, termed Group 1 sRNAs, for which there appears to be an anti-sRNA, ABUWs043 (Weiss *et al*., 2015). ABUWs043 is encoded in an antisense fashion to ABUWs042, and thus may regulate ABUWs042 through several means, including promoter interference and/or complementary binding (Weiss *et al*., 2015). Importantly, ABUWs043 has a high level of sequence complementarity to the rest of the Group 1 sRNAs (21 such elements exist in the *A. baumannii* genome), albeit lower than that found for ABUWs042, suggesting ABUWs043 may regulate the rest of the Group 1 sRNAs in an anti-sRNA fashion (Weiss *et al*., 2015). SAUSA300s206 shares many characteristics with these confirmed and putative anti-sRNAs, but ultimately more work must be done to characterize its function within *S. aureus.* Finally and perhaps the most intriguing observation about these sRNAs is the absence of an identified anti-sRNA encoded in *S. epidermidis* that shares homology with SAUSA300s206. The possibility that such an sRNA exists cannot be excluded, although the potential that this is an *S. aureus* specific adaptation is a potentially fascinating point of evolution. Regardless, a better understanding of the function of these 24 sRNAs may underlie basic physiological and regulatory differences between *S. aureus* and *S. epidermidis*, and further our understanding of the staphylococci in general.

## Supplementary Data

7238 Supplementary File 1

## Supplementary Data

353 Supplementary File 2
