# Supplementary figures and images for: Transcriptomic analysis of staphylococcal sRNAs: insights into species-specific adaption and the evolution of pathogenesis
Source: Microb Genom. 2016 Jul 26;2(7):e000065. doi: 10.1099/mgen.0.000065 (PMC5343137; doi:10.1099/mgen.0.000065)

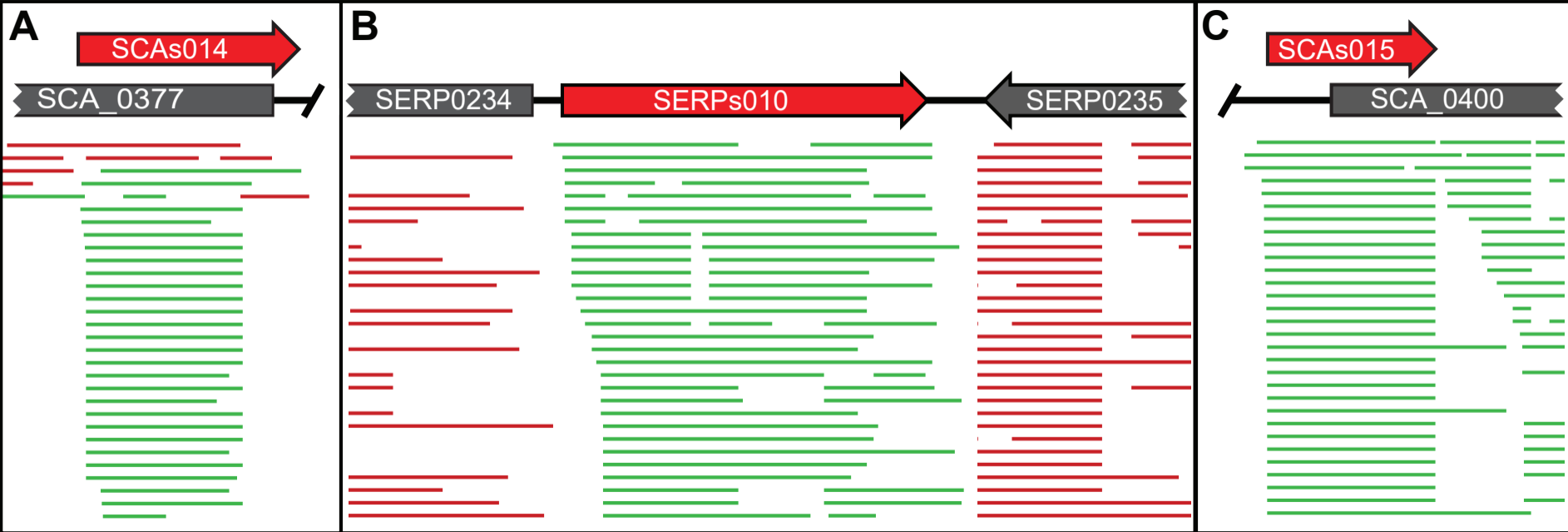



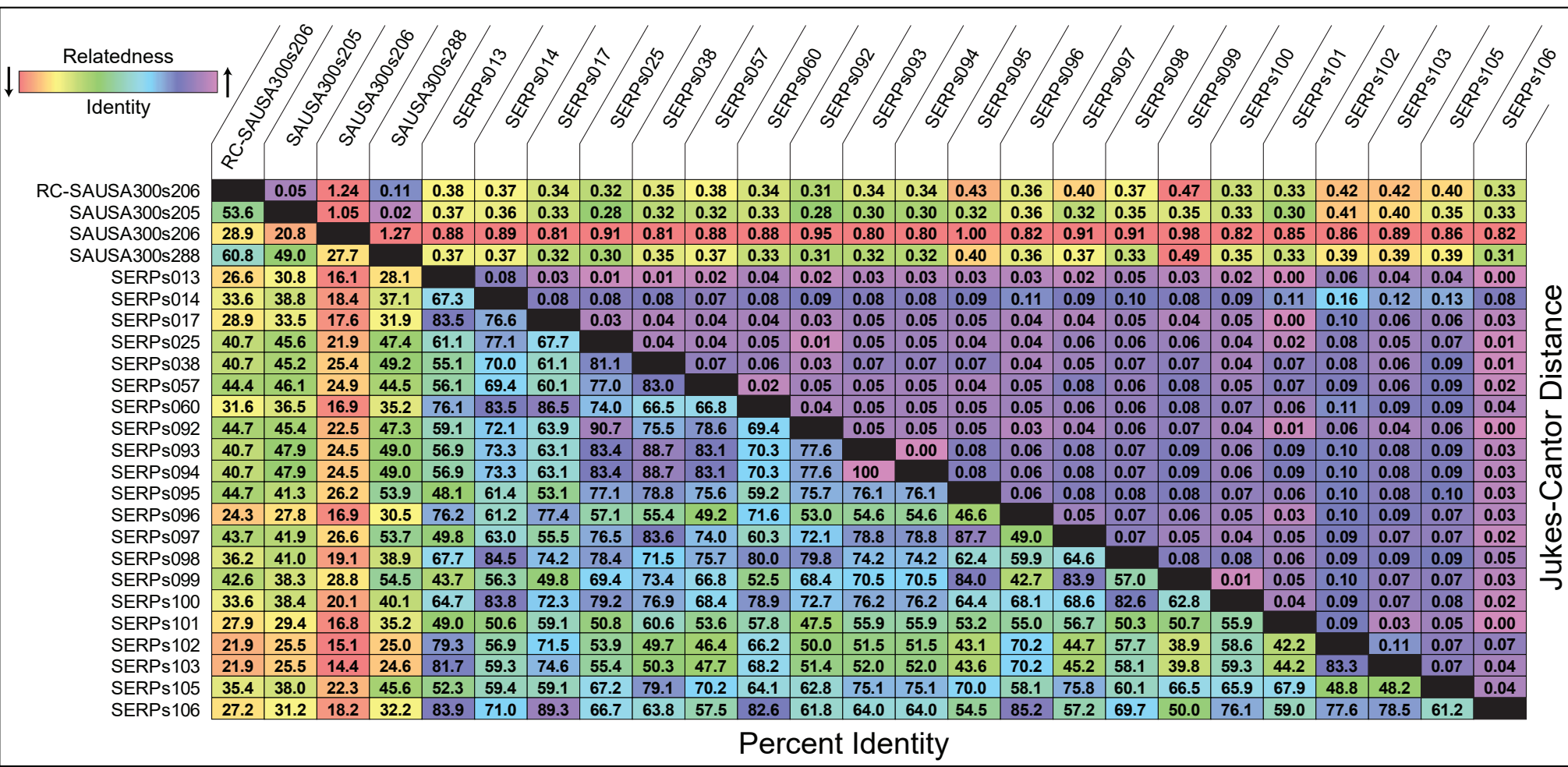

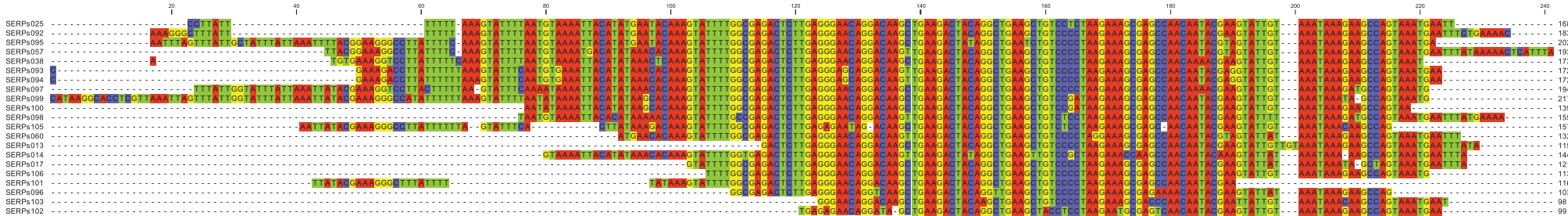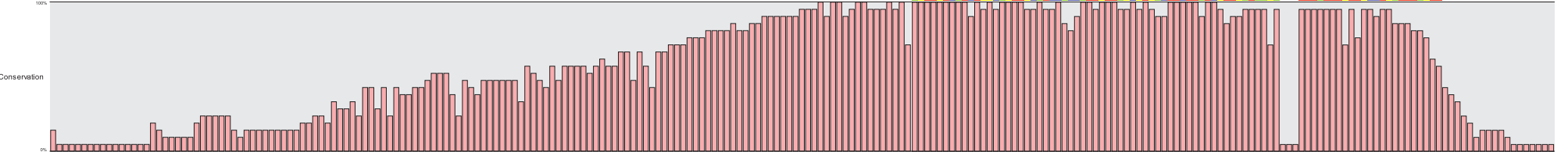

Supplement: Supplementary file 1 [file mgen-02-65-s001.pdf]
